# Peculiar orientational disorder in 4-bromo-4′-nitrobiphenyl (BNBP) and 4-bromo-4′-cyanobiphenyl (BCNBP) leading to bipolar crystals

**DOI:** 10.1107/S2052252516006709

**Published:** 2016-04-28

**Authors:** Matthias Burgener, Hanane Aboulfadl, Gaël Charles Labat, Michel Bonin, Martin Sommer, Ravish Sankolli, Michael Wübbenhorst, Jürg Hulliger

**Affiliations:** a Department of Chemistry and Biochemistry, University of Berne, Freiestrasse 3, CH-3012 Berne, Switzerland; b Laboratory for Soft Matter and Biophysics, University of Leuven, Celestijnenlaan 200d, 3001 Leuven, Belgium

**Keywords:** orientational disorder, bipolar crystals, structural defects

## Abstract

180° orientational disorder of molecular building blocks can lead to a peculiar spatial distribution of polar properties in molecular crystals. Here we present two examples [4-bromo-4′-nitrobiphenyl (BNBP) and 4-bromo-4′-cyanobiphenyl (BCNBP)] which develop into a bipolar final growth state. This means orientational disorder taking place at the crystal/nutrient interface produces domains of opposite average polarity for as-grown crystals. The spatial inhomogeneous distribution of polarity was investigated by scanning pyroelectric microscopy (SPEM), phase-sensitive second harmonic microscopy (PS-SHM) and selected volume X-ray diffraction (SVXD). As a result, the acceptor groups (NO_2_ or CN) are predominantly present at crystal surfaces. However, the stochastic process of polarity formation can be influenced by adding a symmetrical biphenyl to a growing system. For this case, Monte Carlo simulations predict an inverted net polarity compared with the growth of pure BNBP and BCNBP. SPEM results clearly demonstrate that 4,4′-dibromobiphenyl (DBBP) can invert the polarity for both crystals. Phenomena reported in this paper belong to the most striking processes seen for molecular crystals, demonstrated by a stochastic process giving rise to symmetry breaking. We encounter here further examples supporting the general thesis that monodomain polar molecular crystals for fundamental reasons cannot exist.

## Abstract 2

Two dipolar biphenyls show significant 180° orientational disorder resulting in bipolar as-grown crystals. An added symmetrical biphenyl interferes with polarity formation and inverts the initial bipolar state.

## 1. Introduction   

Among many known types of structural defects in crystals, 180° orientational disorder of dipolar molecules can lead to peculiar phenomena when investigating as-grown crystals by new physical methods revealing the *spatial distribution of polar properties* (Hulliger *et al.*, 2002 ▸; Hulliger, Wüst, Brahimi *et al.* 2013 ▸). Taking the benefit of these techniques, a structural analysis of molecular crystals exhibiting significant and spatially variable orientational disorder may thus follow a different strategy than usual in crystallography: In view of the polarization distribution shown in Figs. 5–9, we may ask: For which parts of the crystal is a structural refinement appropriate? Surprisingly, such types of inhomogeneous crystal objects can express a regular morphology (no vicinal, flat faces). Here, a preliminary refinement using data emerging from spatial averaging can be used to initiate a *physical property analysis*, which may be complimented by diffraction data collected from well identified and more or less (property) homogeneous areas in a crystal. In particular, the topological packing and possible strong interaction motifs are the base to assign a possible mechanism of disorder which during growth is producing sectors showing altered polar properties. It is important to notice that the orientational disorder we are addressing here exclusively emerges from the attachment of *incoming molecules* at the crystal/nutrient interface. Once in the bulk, elongated prolate top molecules will not undergo reversal at temperatures far below melting. The analysis presented attempts to understand polarity evolution in 4-bromo-4′-nitrobiphenyl (BNBP) and 4-bromo-4′-cyanobiphenyl (BCNBP) crystals in the frame of a general theory describing *stochastic polarity formation* due to 180° orientational disorder of dipolar molecules (Hulliger *et al.*, 2002 ▸; Hulliger, Wüst, Brahimi *et al.* 2013 ▸).

**Figure 1:**
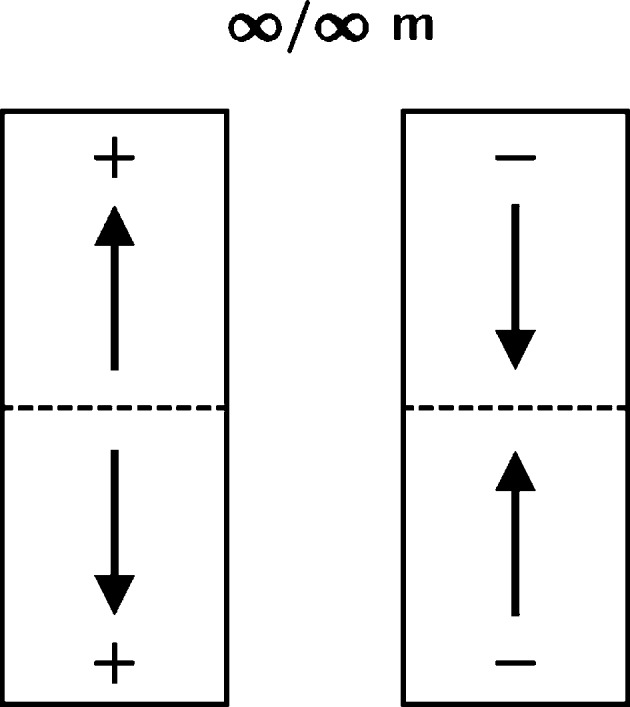
Graphical representation of the two possible bipolar states showing domains of opposite polarity.

Monte Carlo simulations and principles of symmetry breaking at the crystal/nutrient interface allowed us to understand *peculiar* phenomena at first predicted and later found experimentally: The most fundamental fact of relevance to condensed matter showing 180° orientational disorder is that such systems should adopt a *bipolar state* (Hulliger *et al.*, 2002 ▸; Hulliger, Wüst & Rech, 2013 ▸). This means that a crystal built of dipolar entities able to undergo 180° orientational disorder when being attached to a centric or polar seed will develop at least two adjacent sectors showing opposite average polarizations (Hulliger *et al.*, 2002 ▸; Hulliger, Wüst & Rech, 2013 ▸). In case we start from a *centric* seed (*e.g.* *P*2_1_/*c*, 2/*m*), the topological packing of antiparallel dipoles will develop growth-induced faults, which are kinetically stabilized in the bulk. When starting from a *polar* seed the crystal may perform a *reversal transition* (Hulliger *et al.*, 2014 ▸; Burgener *et al.*, 2013 ▸), meaning that along *one* direction of the polar axis, stochastic processes are finally inverting most of the dipoles. Again, as-grown crystals will end up in a bipolar state (see Fig. 1 ▸).

Recently we have been able to demonstrate experimentally this kind of reversal transition (Hulliger *et al.*, 2014 ▸). A most striking phenomenon is observed when solid solutions of (dipolar)_1 − *x*_ (symmetrical)_*x*_ are investigated: The symmetrical molecules undergoing similar synthon interactions induce an *inversion* of the bipolar state, *i.e.* the bipolar state changes the polarization direction in corresponding domains. The systems BNBP and BCNBP are both showing (i) a bipolar state as pure crystals, and (ii) they undergo polarity inversion of the bipolar state when *e.g.* 4,4′-dibromobiphenyl (DBBP) is added to the solution of growth.

The present analysis uses single-crystal X-ray diffraction, scanning pyroelectric measurements to provide a first-step analysis of two rather complex but fascinating molecular crystals, and phase-sensitive second harmonic microscopy for a complimentary identification of opposite polarity in adjacent domains.

## 2. Experimental   

### 2.1. Synthesis and purification of 4-bromo-4′-nitrobiphenyl (BNBP) and 4-bromo-4′-cyanobiphenyl (BCNBP)   

BNBP and BCNBP were produced following published procedures (Le Fèvre & Turner, 1926 ▸; McNamara & Gleason, 1976 ▸). Recrystallization of BNBP was performed from acetic acid by lowering the temperature (pale yellow bipyramidal prisms). Recrystallization of BCNBP was done by sublimation (∼ 150°C) yielding colourless prisms. A GC–MS and MS analysis bears no evidence for impurities such as 4,4′-di­nitrobiphenyl, 4,4′-dicyanobiphenyl or 4,4′-dibromobiphenyl, respectively.

### 2.2. Crystallization methods   

BNBP: (i) Isothermal evaporation of solvent (acetone, acetonitrile, butan-2-one, benzene; 22°C), (ii) lowering the temperature of the solutions (acetic acid, chlorotoluene), and (iii) sublimation in evacuated ampoules (150°C) have all produced bipyramidal prisms. In (ii) rhombic plates were obtained using chloroform and toluene. There is no evidence for polymorphic forms. Melting occurred in the range 175–177°C. All crystals were transparent and colourless. The bipyramidal form expresses faces such as: (110), (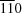), (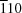), (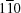), (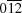), (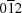), (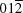) and (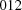). From inspecting the morphology there is no evidence for a polar point group. The rhombic plates feature faces such as: (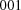), (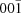), (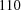), (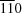), (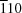), (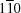). A further small face involving the *b* axis, *i.e.* (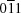), does not show its corresponding (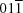) one. Apart from (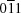), the morphology appears centric.

BCNBP: Here, only (i) isothermal evaporation of solvent (acetone, butan-2-one and toluene) was applied. BCNBP also develops bipyramidal transparent prisms showing the following faces: (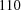), (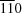), (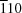), (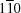), (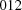), (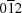), (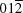) and (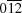).

For second harmonic microscopy, thin melt grown crystals of BNBP, BCNBP were produced. For this, powder was placed in between two glass plates heated above melting. By slow cooling, large single crystalline areas (mm^2^) of a thickness of 20–40 µm were obtained.

To study the influence of symmetrical molecules on the polarity of BNBP, BCNBP, 4,4′-dibromobiphenyl (DBBP) was added to growth solutions of butan-2-one. For comparison and the exclusion of solvents effects, some experiments were also performed in evacuated ampoules.

### 2.3. Single-crystal X-ray diffraction   

Prior to X-ray experiments, all areas of BNBP and BCNBP crystals were visualized by scanning the polarization distribution on the faces of the crystal. The native crystals were glued on a glass fibre with a two-component epoxy resin. Reflections were measured at room temperature using a Stoe Image Plate 2 Diffractometer System (*X-AREA* and *X-RED32* software; Stoe & Cie, 2002 ▸) equipped with Mo *K*α radiation (λ = 0.71073 Å). The intensities were collected in the ω scan mode (scan width ω = 1°) and data reduction was achieved by means of the *X-RED32* program.

At first, full crystals were exposed in order to obtain the metric and structural models. Then, selected volume X-ray diffraction (SVXD) was performed by centring the volumes of interest (about 0.025 mm^3^) into a beam of diameter 0.5 mm.

The structures were solved by direct methods using the program *SHELXS97* (Sheldrick, 1990 ▸) and refined by full-matrix least squares on *F* ^2^ with *SHELXL97* (Sheldrick, 2015 ▸). The H atoms were included in calculated positions and treated as riding atoms using *SHELXL97* default parameters.

The combination of both non-destructive methods SVXD and SPEM allows us to obtain the absolute crystal structure of monodomain volumes with respect to the morphology of crystals even twinned by merohedry (inversion twinning).

### 2.4. Scanning pyroelectric microscopy (SPEM)   

Scanning pyroelectric microscopy (SPEM) allows us to map the spatial polarization distribution of molecular crystals showing a pyroelectric effect (Wübbenhorst *et al.*, 2000 ▸). A change in temperature causes a change of the polarization within such materials. In the case of a small *dT*, the induced current in the capacitor used for measurements is proportional to the absolute value of the polarization *P*. BNBP and BCNBP bipyramidal crystals were polished parallel to the (*b*,*c*)-plane and placed into a capacitor (*b* perpendicular to the electrodes). Additionally, the (*a*,*c*)-plane of a BNBP prism was polished. A modulated laser (λ = 650 nm, 25 mW) reduced to a spot size of less than 10 µm locally heated the surface of the cut plane. The thermally induced current amplified by a Keithley 428 was measured by a lock-in amplifier (Stanford Research SR830).

### 2.5. Phase-sensitive second harmonic generation microscopy (PS-SHM)   

For SHG measurements we used a Q-switched Nd:YAG laser (Surelite I-10, Continuum) providing a repetition rate of 10 Hz with a pulse width of 20–25 ns and a pulse energy of ∼ 25 mJ. This laser generated a fundamental beam at 1064 nm with a pulse intensity of 10 MW cm^2^ and a beam diameter of 4 mm. A Leica polarizing microscope (DM RXP, Leitz) coupled to a dynamic photon counting camera (DynaMight 2000 Camera System, La Vision GmbH) was used in transmission mode. The Leica microscope was additionally joined to a 3CCD colour video camera (DXC-950P Sony). Objectives of 5× and 10× magnifications (LMPLFL Olympus) were used. Single crystals were mounted on glass slides.

## 3. Results and discussion   

### 3.1. Single-crystal X-ray diffraction and morphological analysis   

**Figure 2:**
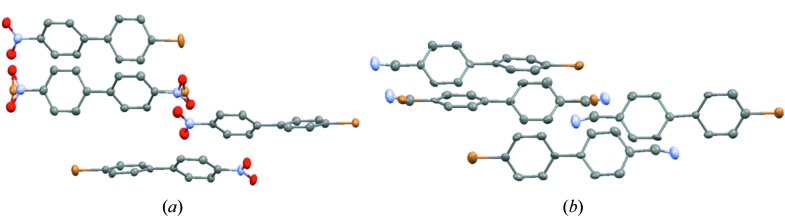
Structural models obtained from full crystal diffraction in the polar space group *P*2_1_. (*a*) BNBP and (*b*) BCNBP (H atoms are omitted for clarity).

Preliminary structures on full crystals of BNBP and BCNBP show molecules arranged along the twofold *b*-axis of the monoclinic cell. Typical synthon interactions —NO_2_⋯Br—, —CN⋯Br— give a topological packing into infinite antiparallel chains. A head-to-tail disorder of NO_2_/Br or CN/Br terminal groups is observed for one chain at least, the carbon skeleton of the biphenyl moiety being undistinguishable. The structures seem to be centrosymmetric but refinements in such a space group did not succeed in stabilizing acceptable models. The best refinements were obtained in the polar space group *P*2_1_ with four independent molecules in the asymmetric unit cell, only one of them showing a head-to-tail 180° disorder (Fig. 2 ▸). Moreover, Flack’s parameter values, around 0.5 [0.49 (3) for BNBP, 0.47 (4) for BCNBP], clearly indicate the presence of an inversion twinning.

A Flack parameter around 0.5 is obviously not adequate to assess the relationship between polarity, morphology and structure. In order to verify the structural model constituted by only one disordered molecule and to clarify the polarity/morphology relationship, the SVXD method was applied to investigate separately the structures of two selected domains on each compound. Due to geometrical constraints to keep the same diffracting volume in the focused beam, the diffraction data are tarnished by some weaknesses (namely for the small crystal of BNBP), *i.e.* completeness, bad resolution, rather high *R*_int_ value, but refined values and agreement factors assess unambiguously the following results: (i) In BNBP, Flack parameters of individual domains (here right and left parts) are found to be 0.06 (4) and 0.10 (4), respectively. The absolute structure is consequently satisfactorily determined. Refined molecule populations for the asymmetric unit result in a 1.584 (4) bromine group toward the +*b* direction, 2.416 (4) toward −*b* for the right part, and a 2.361 (6) bromine group toward +*b* direction, 1.639 (6) toward −*b* for the left part. (ii) In BCNBP Flack parameters of each domain, *i.e.* top and bottom, values probing here again the monopolar character of the selected volumes are respectively refined to 0.085 (25) and 0.061 (19). For the top-volume, the refined molecule populations of the bromine group in the asymmetric unit give a population of 2.293 (4) toward the +*b* direction and 1.707 (4) toward the −*b* direction. In the case of the bottom-volume investigated, we find a 1.539 (3) bromine toward the +*b* direction, 2.461 (3) toward the −*b* direction.

With all these results the relationship between the bipolar crystal morphology and the absolute structure can be established: In both crystal systems the acceptor groups (NO_2_, CN) are pointing in a predominant way from inside the crystal to outside, *i.e.* towards the growing interface.

From the van der Waals shape of BNBP and BCNBP molecules undergoing rather a densely packed structure we can conclude that this kind of orientational disorder is not a bulk equilibrium property. The activation for dipole reversal in the bulk would by far be too high at room temperature. Therefore, this disorder is taking place at the crystal-to-nutrient interface. Evidently its extension is influenced by the interplay of solvation and attachments of molecules to growing faces.

Following the approved theory of *growth-induced polarity formation*, the growth of a seed will be associated with orientational disorder and thus symmetry-allowed crystal sectors (Hulliger *et al.*, 2002 ▸; Hulliger, Wüst & Rech, 2013 ▸) can build up a vector-type property, *i.e.* a pyroelectric effect.

As mentioned during the introduction both a centric and a polar seed can end up in a bipolar state, in other words, can represent 180° non-classical twins. For this we have investigated the spatial variation of the Flack parameter (see supporting information).

Summarizing X-ray data and physical measurements, we can assign a polar structure to sectors involving the *b*-axis. The only likely space group is *P*2_1_ (2). In case the reader accepts that in BNBP and BCNBP orientational disorder is a grown-in phenomenon, we are at the point of raising a basic question: What is the symmetry of the seed? Following the most recent results (Hulliger *et al.*, 2002 ▸; Hulliger, Wüst & Rech, 2013 ▸) the seed in a stationary state should be bipolar as well. However, nucleation is usually taking place at rather high supersaturation and thus kinetic control may lead to a (i) centric or (ii) polar seed. Here, averaged (several hundred µm) X-ray data do not provide further help. In case we have a clear situation of (i) a centric seed followed by growth-induced polarity formation, symmetry-related sectors show a similar polarity distribution. Otherwise (ii) a polar seed may undergo a reversal transition also leading to a similar polarity distribution in corresponding sectors after the transition (macroscopic zone; Hulliger *et al.*, 2002 ▸; Burgener *et al.*, 2013 ▸) is over. Here, pyroelectric data seem to demonstrate that BCNBP is rather following case (i), *i.e.* centric seed, whereas BNBP is likely to undergo mechanism (ii), *i.e.* polar seed followed by a reversal transition (Figs. 6 and 7).

### 3.2. Phase-sensitive second harmonic generation analysis   

**Figure 3:**
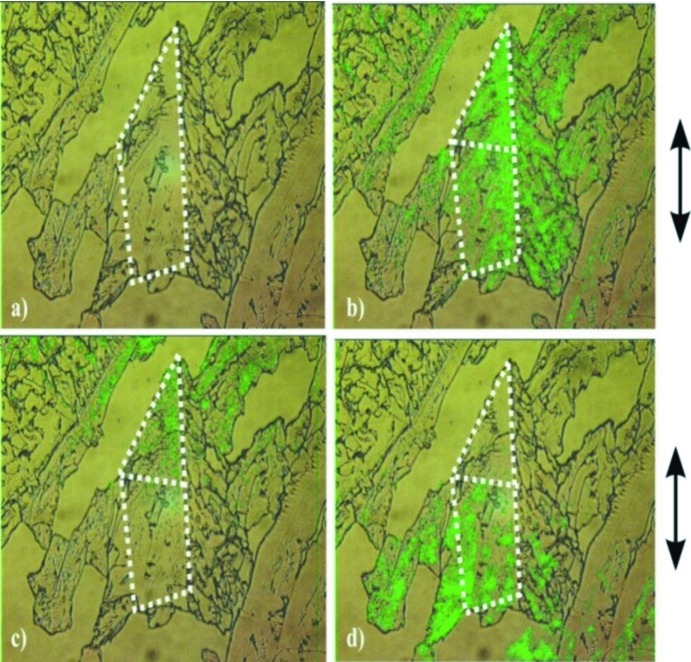
Second harmonic generation analysis to show polarity formation in micrometer thin crystal plates of 4-bromo-4′-nitrobiphenyl (BNBP) grown from the melt. White dotted line: central area where growth occurred. (*a*) Linear optical image of the crystal surrounded by a white dotted line. (*b*) Presence of polarity (dark green) in both the upper and lower sectors. (*c*) and (*d*) effect of phase contrast for the lower and upper sector.

Preliminary to the analysis by SPEM we performed edge-defined thin film melt growth for both BNBP and BCNBP. A further goal was to compare solution, melt and vapour growth results because of the known effects of solvents (Behrnd *et al.*, 2010 ▸) on the growth of polar crystals. In Figs. 3 ▸ and 4 ▸ we present (i) a microscopy image (*a*) and (ii) SHG results (*b*)–(*d*). Because of intergrown parts, the analysis is restricted on the parts that are marked (white broken lines).

By turning the crystal at a fixed polarization of the laser, the direction of maximum response was found. At 90° to this orientation no signal appeared, meaning that the β_zzz_ hyperpolarizability axes of aligned molecules are perpendicular to the polarization of the ω_0_ light. Figs. 3 ▸ and 4 ▸ show an inhomogeneous response, presumably along the *b*-axis. The spatial variation of the response stems mainly from an inhomogeneous polarity distribution then effected by a sample thickness being larger here than the coherence length. The phase-sensitive experiments (*c*,*d*) let us localize the seed region, because of the appearance of two domains featuring opposite polarities. These PS-SHM experiments allow us only to conclude that (i) significant 180° orientational disorder is present, (ii) the disorder varies in space, (iii) a bipolar growth state is obtained and (iv) the phenomenon is also present at high temperature, *i.e.* for growth upon a supercooled melt. No further distinction on the possible polar state of the seed can be made here.

## 4. Scanning pyroelectric microscopy analysis   

SPEM measuring the local pyroelectric response of a material provides a map of the polarization distribution in BNBP and BCNBP crystals. Because *P* is mainly emerging from a sum over the polarity of individual molecules (modified by the effect of their surrounding molecular field), *P* is proportional to the sum of weighted SOF values: *P* = ε(SOF(Br1)α_1_ + SOF(Br2)α_2_ + SOF(Br3)α_3_ + SOF(Br4)α_4_), where α_*i*_ (*i* = 1,…, 4) are factors taking into account (i) the inclination of μ_mol_ with respect to axis 2, and (ii) the modification of μ_mol_ by its surrounding, *ε* being a conversion factor. Depending on the degree of orientational disorder, the spatial response *P*_(*x*,*y*,*z*)_ may vary in between *P*_max_ (all μ_*i*_ pointing in the same direction) and *P* = 0 (centric structure).

**Figure 5:**
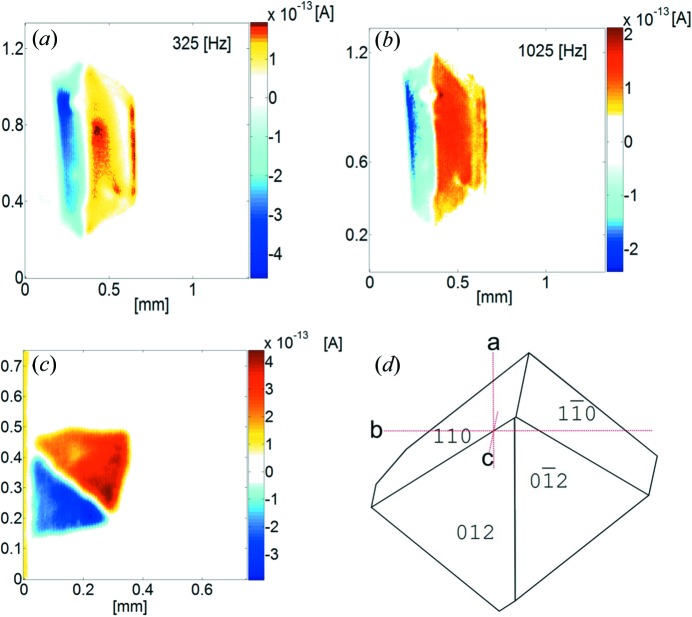
Polarity distribution for BNBP: SPEM measurements for a polished (*b*,*c*)-plane (*a*) 325 Hz, (*b*) 1025 Hz). (*c*) SPEM measurement at 325 Hz for a polished (*b*,*a*)-plane. (*d*) Morphology of a BCNBP crystal.

BNBP: Scanning the (*b*,*c*)-plane of two polished crystals we find two parts of obviously opposite polarity (Fig. 5 ▸ *a*). This was obtained by measuring the direction of the discharge current. Assuming a negative effective pyroelectric coefficient, on average NO_2_ groups are oriented towards the nutrient. The spatial variation becomes evident (right side) when changing the frequency of the modulated heat source: At lower frequency (325 Hz, Fig. 5 ▸ *a*) a different degree of net polarization and distribution is obtained compared with 1025 Hz (Fig. 5 ▸ *b*). At low frequency the penetration depth is larger than at high values (Wübbenhorst *et al.*, 2000 ▸).

**Figure 6:**
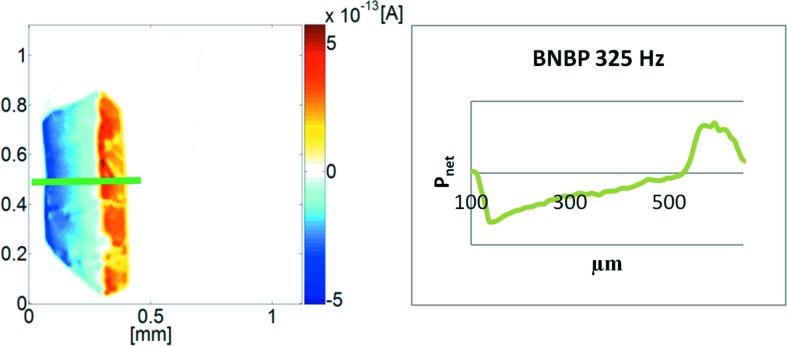
BNBP SPEM measurement at 325 Hz for a polished (*b*,*c*)-plane. Green: The normalized polarization of the transition zone located between two mono-polar domains is measured: The polarization is continuously changing from minus to plus over a distance of ∼ 500 µm.

Measuring a second crystal prepared for scanning the (*b*,*c*)-plane revealed a similar behaviour. From these measurements we can conclude: BNBP as a seed (Fig. 6 ▸) develops into *two domains of opposite polarity*, whereas one of them seems to show more inhomogeneities (right, Fig. 5 ▸ *a*) than the other. However, there are additional defects, visible in optical microscopy. The *nitro-groups* are preferentially pointing towards the growing interface (as confirmed by X-ray).

The transition from one domain into the adjacent one is rather broad (Fig. 6 ▸), *i.e.* much larger than previously found for 4-iodo-4′-nitro­biphenyl (INBP ∼ 150 µm), representing a polar structure with nearly full parallel alignment of dipoles (*Fdd*2, *mm*2; Burgener *et al.*, 2013 ▸).

In view of these results, we propose here that BNBP seeds are *polar* and the growing crystal is undergoing a *reversal transition*. Because acceptor (NO_2_) groups are preferably covering the surfaces in the ±*b* directions of the bipolar object, stochastic theory of polarity formation (Hulliger *et al.*, 2002 ▸, 2014 ▸) predicts here that the *donor* side of the polar seed undergoes a *reversal transition*. The absolute structure achieved by our X-ray studies confirms such a mechanism.

**Figure 7:**
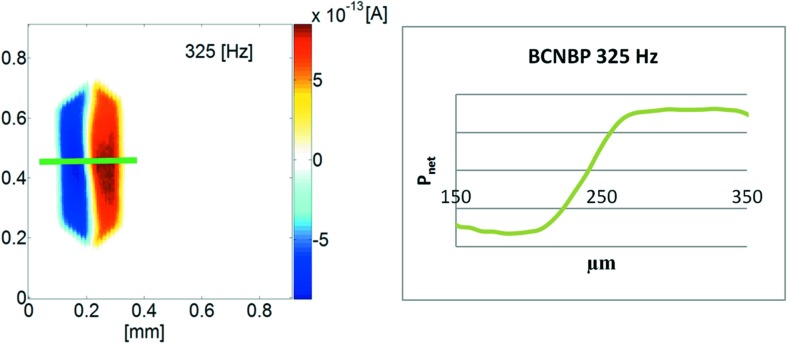
SPEM measurement at 325 Hz for a polished (*b*,*c*)-plane of a BCNBP crystal. Green: The normalized polarization of the transition zone between two mono-polar domains shows a much smaller width compared with BNBP.

BCNBP: In comparison to BNBP, this material shows a rather constant and equally strong polarization in both domains of a bipolar state. Also here the acceptor (CN) groups preferably cover the polar faces (Fig. 7 ▸). So, most likely a non-polar seed is generated, which develops into a bipolar state.

**Figure 8:**
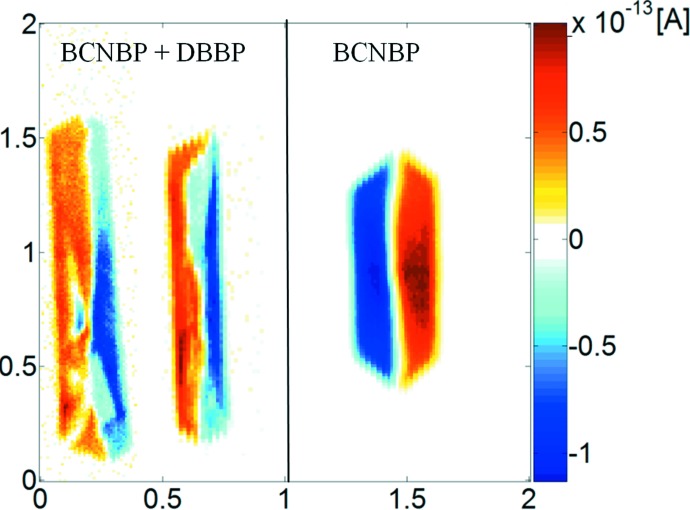
Left: Solid solution of BCNBP and DBBP. SPEM measurement at 325 Hz for a polished (*b*,*c*)-plane (results for two different crystals are shown). Right: SPEM measurement at 325 Hz for a polished (*b*,*c*)-plane of a *pure* BCNBP crystal. SPEM reveals two different bipolar states: left: ←→, right: →←.

A key experiment for an experimental confirmation of polarity formation due to orientational disorder can be set up from the following theoretical prediction: Monte Carlo simulations of polarity evolution in solid solutions (*A*–π–*D*)_1 − *x*_ (*D*–π–*D*)_*x*_ predict (Roth *et al.*, 1998 ▸) an *inverted final state* for a system *A*–π–*D* undergoing a reversal transition. This means that a significant addition of *D*–π–*D* to the nutrient can reverse the polarity of the bipolar state without significant uptake of *D*–π–*D* by solid solution formation. SPEM investigations of BNBP and BCNBP crystals grown in the presence of DBBP (4,4′-dibromobiphenyl) revealed clearly an *inverted* situation in both cases (Fig. 8 ▸). These experiments with solid solutions provide clear evidence that this 180° domain formation is not established *via* a classical twinning mechanism.

**Figure 9:**
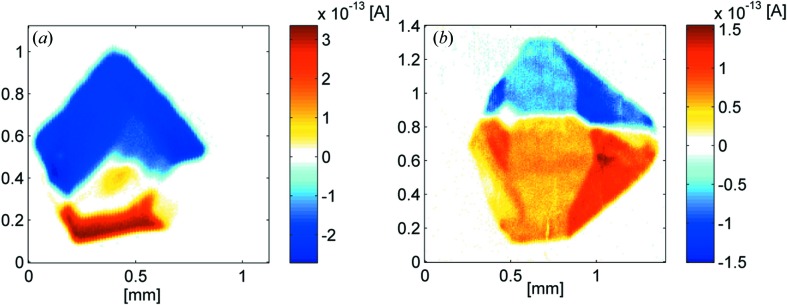
Crystals grown from vapour: SPEM measurement at 325 Hz for a polished (*b*,*c*)-plane of (*a*) a BCNBP and (*b*) a BNBP crystal. This result using vapour grown crystals demonstrate that the solvents used for other experiments may well have an influence, but they cannot be made responsible for the main effect of polarity formation and the reversal transition.

In addition to results from *solution* and *melt* grown crystals, a bipolar state has been found for BNBP and BCNBP crystals grown from *vapour* (Fig. 9 ▸). Basic effects of solvents on the growth of polar crystals can thus be excluded here, *i.e.* the observed phenomenons are not due to effects of solvents.

## 5. Summary and conclusions   

Solution and vapour grown BNBP and BCNBP crystals can express a regular and symmetrical morphology featuring flat faces, despite representing inside a twinned, *i.e.* bipolar, object featuring an inhomogeneous polarization distribution. Topologically, the packing may be described by antiparallel chains formed by four independent molecules. Polarity emerges from orientational disorder on one site, which is not a feature of a minimum energy bulk structure, but the result of a process at the *growing interface*. Although lattice sums have to be taken into account, a rather simple synthon interaction scheme (Hulliger *et al.*, 2002 ▸; Burgener *et al.*, 2013 ▸) can explain (i) sectorwise polarity formation, and (ii) which functional group is preferentially oriented in the direction of the polar *b* axis: In both structures the NO_2_⋯Br and the —CN⋯Br— (*A*⋯*D*) interactions can be considered as strong short-range contacts. Reversal at the surface involves —Br⋯Br— (*D*⋯*D*) and —NO_2_⋯O_2_N— or —CN⋯CN— (*A*⋯*A*) contacts, where —Br⋯Br— may show a local energy minimum and —*A*⋯*A*— is destabilizing. Consequently, we have (i) *E*_DD_ − *E*_AA_ ≠ 0, and most likely (ii) *E*_AA_ > *E*_DD_. The first condition ensures that polarity can evolve, the second is giving preference for *A*-groups at the surface. The way in which BNBP and BCNBP crystals develop into a bipolar state may allow us to distinguish between a (i) *centric* and a (ii) *polar* seed: The rather large transition zone of BNBP (Fig. 6 ▸) may be due to a seed which by kinetic control is *polar*. The sharp transition found for BCNBP may be due to *a centric or a bipolar seed*, developing into a bipolar final state. The most striking phenomenon, however, is the observation of an *inversed* bipolar state, when adding *D*–π–*D* molecules to the nutrient. Instrumental analysis has traced very little of *D*–π–*D* in terms of a solid solution (*A*–π–*D*)_1 − *x*_ (*D*–π–*D*)_x_, *i.e.* the distribution coefficient is low. Here we identify a lack of understanding, because MC simulations for model systems also predicted a low distribution coefficient, but obviously larger than found experimentally.

The present results end up in a first step analysis. From here, deeper crystallography (diffuse scattering analysis) may investigate volume parts of DNBP or DCNBP crystals being well characterized by SPEM, with the aim of disclosing more about the peculiar disorder of such types of disordered crystalline states.

## Supplementary Material

Crystal structure: contains datablock(s) BNBP_left, BNBP_right, BCNBP_bottom, BCNBP_top. DOI: 10.1107/S2052252516006709/bi5055sup1.cif

Structure factors: contains datablock(s) shelxl. DOI: 10.1107/S2052252516006709/bi5055BNBP_leftsup2.hkl

Structure factors: contains datablock(s) shelxl. DOI: 10.1107/S2052252516006709/bi5055BNBP_rightsup3.hkl

Structure factors: contains datablock(s) shelxl. DOI: 10.1107/S2052252516006709/bi5055BCNBP_bottomsup4.hkl

Structure factors: contains datablock(s) shelxl. DOI: 10.1107/S2052252516006709/bi5055BCNBP_topsup5.hkl

Supporting information file. DOI: 10.1107/S2052252516006709/bi5055BNBPsup6.cml

Supporting information file. DOI: 10.1107/S2052252516006709/bi5055BCNBPsup7.cml

CCDC references: 1475349, 1475350, 1475351, 1475352

## Figures and Tables

**Figure 4:**
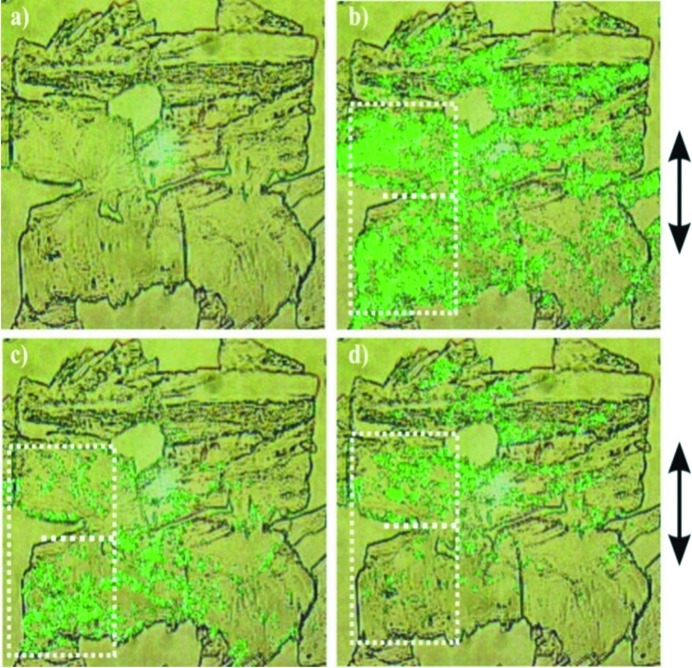
Second harmonic generation microscopy in transmission for a melt grown crystal of BCNBP. White dotted line: central area where growth occurred. Green: areas where growth induced polarity developed. (*a*) Linear optical image. (*b*) Two sectors show polarity. (*c*) A phase-sensitive experiment showing an SHG effect only for the upper sector. (*d*) Same as (*c*), but sample turned around 180°.
